# SIT-ia: A Software-Hardware System to Improve Male Sorting Efficacy for the Sterile Insect Technique

**DOI:** 10.3390/insects16111108

**Published:** 2025-10-30

**Authors:** Gerardo de la Vega, Luciano Smith, Lihuen Soria-Mercier, Wilson Edwards, Federico Triñanes, Santiago Masagué, Juan Corley

**Affiliations:** 1 Instituto de Investigaciones Forestales y Agropecuarias de Bariloche, Instituto Nacional de Tecnología Agropecuaria–Consejo Nacional de Investigaciones Científicas y Técnicas (IFAB, INTA–CONICET), San Carlos de Bariloche 8400, Río Negro, Argentina; 2 Facultad de Ingeniería, Universidad de Buenos Aires (UBA), Buenos Aires C1063ACV, Argentina; 3 Centro Regional Universitario Bariloche, Universidad Nacional del Comahue CRUB (UNCOMA), San Carlos de Bariloche 8400, Río Negro, Argentina; 4 Servicio Nacional de Sanidad y Calidad Agroalimentaria (SENASA), Centro Regional Patagonia Norte, San Carlos de Bariloche 8400, Río Negro, Argentina; 5 Laboratorio de Ecología Química, Facultad de Química, Universidad de la República (UdelaR), Montevideo 11800, Uruguay

**Keywords:** pest management, male sorting, spotted wing drosophila, machine learning, artificial intelligence, convolutional neural network architectures

## Abstract

Invasive insects can cause significant economic impacts to agriculture worldwide and impact human health. Traditional pest management methods that include chemical insecticides have raised increasing environmental and health concerns, prompting the need for sustainable alternatives. The Sterile Insect Technique (SIT), which consists of releasing sterile males of a target pest to mate with wild females, is held as a promising solution. However, the success of SIT relies on the release of sterile males. The efficient separation of sexes prior to sterilization and release is necessary. This study presents SIT-ia, a software–hardware system that utilizes artificial intelligence (AI) and computer vision to automate the sex-sorting process. We showcase its use with the fruit fly pest *D. suzukii*. The system was able to identify males from females with a 98.6% accuracy, sorting 1000 sterile flies in ~70 min, which is nearly half the time involved in manual sorting by experts (i.e., ~112 min). This simple device can easily be adopted in SIT production protocols, improving the feasibility and efficacy of improved pest management practices.

## Simple Summary

This research addresses a challenge in using the Sterile Insect Technique (SIT), an eco-friendly pest control method. For SIT to work, only sterile male insects can be released, but sorting males from females by hand is slow and laborious. The study introduces a new automated system called SIT-ia that uses artificial intelligence (AI) to quickly and accurately tell male and female flies apart. When tested on the spotted-wing drosophila (*Drosophila suzukii*), the system was 98.6% accurate. A key benefit is its speed: SIT-ia can sort 1000 flies in about 70 min, which is 40 min faster than human experts. This innovation makes sex-sorting a more efficient and practical process, needed for managing pest insects in a sustainable way.

## 1. Introduction

Herbivorous alien insects increasingly threaten ecosystems, food production, and human well-being worldwide. This is because they can establish novel associations with host plants that favor rapid population growth in the invaded range, often becoming severely damaging pests that require control measures [1]. The overall aim of invasive pest management protocols is to eradicate them during the early stage of invasion or push established populations toward low densities so that population growth becomes increasingly unlikely due to factors such as the inability to find mates and a reduced capacity to avoid natural enemies or to successfully exploit food resources, together with an increased exposure to demographic and environmental stochasticity [2,3,4].

Leading invasive pest populations toward an eradication threshold or preventing reestablishment are guiding principles of the Sterile Insect Technique (SIT) of pest management. Also, the technique is used to create areas of low pest prevalence for established populations. The SIT is regarded as an environmentally friendly approach against a variety of pests because it focuses on the harmful species without affecting the rest of the insect community. As such, it has been successfully applied to a variety of pests, such as the screw-worm fly (*Cochliomya hominivorax* (Coquerel, 1858)), several tephritid fruit flies (*Ceratitis capitata* (Wiedemann, 1824), *Anastrepha ludens* (Loew, 1873), *Bactrocera cucurbitae* (Coquillett, 1899), etc.), some species of tsetse flies (*Glossina* spp.), and the pink bollworm (*Pectinophora gossypiella* (Saunders, 1844)) [5]. More recently, the SIT is being explored to manage damaging populations of the spotted wing drosophila (SWD, *Drosophila suzukii* (Matsumura, 1931); Diptera: Drosophilidae) with encouraging results [6,7].

The SIT involves three steps: first, the mass rearing of the insect pest in specialized and secure facilities; then, exposing specimens to ionizing radiation (gamma or X-rays) to sterilize them; and finally releasing the sterile males only into the area where a wild population is established. The concept behind this approach is that the release into the environment of sterile adult males to mate with wild females will negatively impact population growth because females that have mated with sterile males will lay infertile eggs. To achieve these objectives, the density of the released sterile males must be higher than that of wild males (i.e., for mosquito SIT trials, a release ratio of 10:1 is generally the minimum needed [8]). It is also necessary that released males do not suffer a reduction in their mating competitiveness compared to wild males [9].

Generally, the release of both sterile males and females in SIT programs is regarded as costly and less effective because sterile females do not contribute to population suppression and may compete with males for mates [4]. However, for some species such as SWD, male-only releases are essential for success, as released females, even if sterilized, can damage crops attempting to lay eggs. For vector species like mosquitos, sterile females are also problematic, as they still may feed on humans and contribute to disease transmission [5]. In consequence, in most SIT protocols, efficient sex separation methods, such as genetic, mechanical or manual, or fluorescence-based systems, are essential to improve the effectiveness of the technique [10,11,12,13].

Recently, the application of deep learning methods has emerged as a powerful tool for the automatic identification of insects. Through this approach, insects are usually trapped on sticky surfaces, which are then photographed and analyzed. Insects are then counted and identified using simple learning algorithms, computer vision, and artificial intelligence (AI) [14,15,16]. A recently studied aspect is the use of AI in SIT for the determination of the physiological age of pupae (for example, *A. ludens* and *C. capitata*) from images to reduce human error and uncertainty in irradiation timing [17]. However, despite their unquestioned potential, such novel tools and techniques have not been extensively explored, needing species-specific adaptation and validation, especially if they are to be widely implemented in SIT programs.

Here, we describe an AI-assisted computer-based vision system for the sex differentiation of specimens to be used in SIT. Our hardware–software system allows for the sexing and also the killing of females, which we test with *D. suzukii* as a case model, comparing processing times against manual sex-sorting. We present a simple device that may be readily adopted in SIT protocols, improving the feasibility and efficacy of this protocol for improved sustainable pest management practices.

## 2. Materials and Methods

### 2.1. Drosophila suzukii Colony

The insect culture held in our laboratory (IFAB INTA-CONICET) provided flies for the development of the SIT-ia and testing. The *D. suzukii* colony was established in 2022 from wild flies collected in the locality of El Hoyo (Chubut province, Argentina). The flies were maintained in 30 × 30 × 30 cm cages, with an average population size of approximately 3000–4000. The rearing conditions were 12 h L:D photoperiod, 21 °C (±1 °C), and 65% RH in a cornmeal media containing 0.8% agar, 4% baker’s yeast, 8% corn flour, 10% glucose (*m*/*v*), 0.3% propionic acid, and 0.9% (*v*/*v*) nipagin 10% (*m*/*v*) solution, expressed as a function of the amount of distilled water.

### 2.2. Classification Algorithms

The analysis of acquired images was performed using a two-stage pipeline. The first stage, detection, involved processing the full image to identify and localize every individual *D. suzukii*, returning a count and coordinates for each. The second stage, classification, involved extracting each detected individual based on its coordinates and passing the resulting image crop through a deep neural network to assign a sex label to it (male or female). The term “detection” throughout this manuscript refers solely to the counting and localization of individuals, which is a prerequisite for the sex classification task.

To classify sexes of our studied *D. suzukii* adults, we used six neural network architectures based on the work of Bischel [18]. Architectures used were as follows: (1) VGG16, developed by the Visual Geometry Group (VGG), is a simple yet effective network for image classification. It consists of 16 layers, 13 of which are convolutional and 3 are fully connected [19]. (2) VGG19 is similar to VGG16, but comprises five blocks of sixteen convolutional layers, ending with a sixth block of three fully connected layers [20]. (3) ResNet 50, ResNet 101, and ResNet 152 are three deep neural network architectures with 50, 101, and 152 layers, based on Microsoft’s ResNet architecture [21]. ResNet introduces residual learning blocks, mitigating the vanishing gradient problem and enabling more efficient training of deep networks. (4) MobileNet V2, developed by Google, is a convolutional neural network designed for mobile devices. Its key features are inverted residuals and linear bottlenecks [22]. (5) EfficientNet is another Google-developed convolutional neural network that optimizes computer vision tasks like object classification. It balances depth, width, and resolution scaling to achieve high precision with fewer computational resources [23]. (6) Finally, we used a neural network architecture named CNN6, consisting of six layers: the first four were convolutional layers with Rectified Linear Unit (ReLU) activation, followed by max-pooling layers to reduce dimensionality, and the last two were fully connected layers. See Table 1 for architectural characteristics of the deep learning models used for classification.

### 2.3. Software Design and Hardware Used

We employed a combination of software and hardware to develop the automated system. The software was built using Python (v3.12) [24] as the primary language, leveraging several key libraries: Pandas [25] for data processing, OpenCV [26] for image-based insect detection and capture, and PyTorch (v2.7) [27] for implementing the deep learning-based sex classification neural networks. Machine instructions for laser control were generated using GCode [28] to ensure precise movement and operation. The software was developed on Windows 11 Pro system (the computer was powered by an AMD Ryzen 7 5700G processor and utilized an NVIDIA GeForce RTX 3070 Ti GPU (8 GB VRAM) to accelerate the training and inference of the deep learning models, equipped with 32 GB of RAM to handle in-memory data processing). Finally, the SIT-ia can run on Windows or Mac laptops using two USB ports and Python > v3.10 as main requirements.

The hardware platform consisted of three integrated components: the Image Acquisition System, a Laser Elimination System, and an Anesthesia System. For high-resolution image capture, we used a LapSun 12 MP industrial camera (4000 × 3000 pixels, Lapsun, Shenzhen, China) with a “C” mount. This was fitted with a 16 mm HTENG VISHI lens (model HTFA1611A, Shenzhen Huateng Vision Technology, Shenzhen, China) and a ring light to provide consistent, shadow-free illumination for sharp image quality (Neje Laser tools, Shenzhen, China). Secondly, to target and eliminate specific specimens, a Neje Master 2 7W laser module was selected. This laser met the required specifications for precision, speed, and a power of 7 W, which was determined to be effective through preliminary testing. The use of a laser requires the operators to wear eye protection glasses (see user manual [29]). The module features a GCODE-compatible controller with USB connectivity, allowing for seamless operation and control directly from our Python software. Finally, flies were immobilized for processing using a Flystuff anesthesia plate (10.1 × 14 cm Genesee Scientific, San Diego, CA, USA) supplied with CO_2_.

### 2.4. Route Optimization

Image processing allows identification of sterile males from females. The latter are killed by means of a laser beam that needs an optimized pathway to avoid increased processing times and mechanical wear associated with unnecessary directional changes. To optimize the path, three algorithms were tested, selectable via a graphical interface. The problem of finding the shortest path covering all points is known as the Traveling Salesman Problem (TSP). Exact solutions are computationally expensive, so three heuristic algorithms were used and tested against unoptimized routes. First, the Greedy Algorithm was tested: this step-by-step approach starts at the laser’s initial position, moving to the nearest insect and repeating the process, avoiding revisits. While not globally optimal, it provides efficient, approximate solutions quickly [30]. Second, the Local Search Algorithm was tested: this method iteratively improves an initial solution by exploring neighboring solutions. Though prone to local optima, it outperforms the Greedy approach [30]. Finally, we tested Ant Colony Optimization (ACO), which is inspired by the foraging behavior of ants. ACO models the problem as a graph where edges (paths) have “pheromone” levels guiding “ants” toward optimal routes. Solutions are constructed probabilistically, with pheromones updated based on solution quality [30].

### 2.5. System Performance

Evaluation of the sex-sorting performance was conducted by comparing it with manual sorting by trained staff. The “experts” were some of the authors of this manuscript (GD, LS, WE, FT, and SM) who have worked on SWD for more than three years. Flies used were gently collected from the colony by aspiration (100–300 individuals) and transferred to 250 mL plastic vials containing ~10 mL of sugar–agar media, until preparation by the experts. Time expended between preparation and sex-sorting and different strategies (stereomicroscopy or simple vision) of the experts was also registered. Time required to analyze 1000 specimens of *D. suzukii* was compared by statistical analyses performed in R v4.3.1 [31].

### 2.6. Model Explainability

Despite the model’s high performance in classifying sexes in tested flies, understanding the specific reasons behind its decisions remained challenging. Neural network decision-making is often perceived as a “black box” [32]. Identifying the reasons behind classifications and factors contributing to errors is crucial for optimizing model performance. Explainability studies can also reveal new insights, such as additional morphological features distinguishing male and female insects, fostering user trust and collaboration in model improvement.

However, interpreting neural network decisions is limited, prompting the development of explainability studies. In this work, the RISE (Randomized Input Sampling for Explanation of Black-box Models) technique [33] was selected to investigate model interpretability. RISE subjects the model to multiple randomly masked versions of an image, calculating weighted averages of all masks based on their scores to generate a final heatmap. The premise is that masks preserving relevant image features receive higher scores and greater weight in the average. For the model analysis using RISE, 10,000 randomly masked versions were generated for each image: one male from both dorsal and lateral views, and one female from both dorsal and lateral views.

## 3. Results

### 3.1. Classification Algorithms

First of all, to determine the algorithm’s performance for the detection of specimens, 100 images of the plate, each with about 90 live individuals, were used (n = 100). The detection accuracy was visually verified, confirming the correct insect identification. Out of 9216 detections, only 49 were incorrectly sorted, resulting in a 0.53% error rate. A detailed analysis revealed that all errors were due to a stain on the anesthesia plate surface. This stain caused two types of errors: (1) increasing the detected surface area beyond the maximum threshold and (2) being misinterpreted as a valid fly when separated from insects. Although adjusting the minimum and maximum thresholds was tested (configurable via a user-friendly interface), modifying these thresholds is not recommended, as it reduces the classification accuracy for other insects. Users can manually correct detection errors and remove as many potential error sources as possible.

A subset of 3272 detected flies was curated to create a sex-balanced dataset for training and evaluating the classification models. The data were split into training (60%), validation (20%), and test (20%) sets, maintaining an equal number of males and females in each partition. This resulted in 1964 images (982 per sex) for training and 654 images (327 per sex) each for validation and testing. The classification algorithm results showed that VGG16 achieved very good results but was not the top-performing architecture. The VGG19 architecture achieved a slightly lower accuracy than VGG16, indicating that the increased complexity did not translate to better results in this case. For ResNet 50, ResNet 101, MobileNet V2, EfficientNet, and ResNet 152, none of these three architectures outperformed VGG16. However, CNN6, a simple convolutional neural network, outperformed the more complex architectures tested earlier and was chosen (Table 1).

### 3.2. Software Design and Implementation

After building a test prototype, a dataset with 8518 *D. suzukii* images was generated using the image acquisition equipment. Of these, 4799 (56%) were female and 3719 (44%) were male. To balance the dataset, 1080 male images were augmented with random rotations (90° clockwise or counterclockwise), resulting in a balanced dataset of 9598 images. The data was split as follows: 60% for training (5760 images), 20% for testing (1379 images), and 20% for validation (1379 images). The CNN6 model was trained in two modes: using transfer learning (pre-trained on the development dataset) and from scratch. Transfer learning did not improve the results, so training from scratch was used. The final training achieved a 98.08% accuracy on the validation set and 98.94% on the training set after 200 epochs, with a total processing time of 586 min. However, the training curves became asymptotic after epoch 160, showing no further improvement.

**Figure 1:**
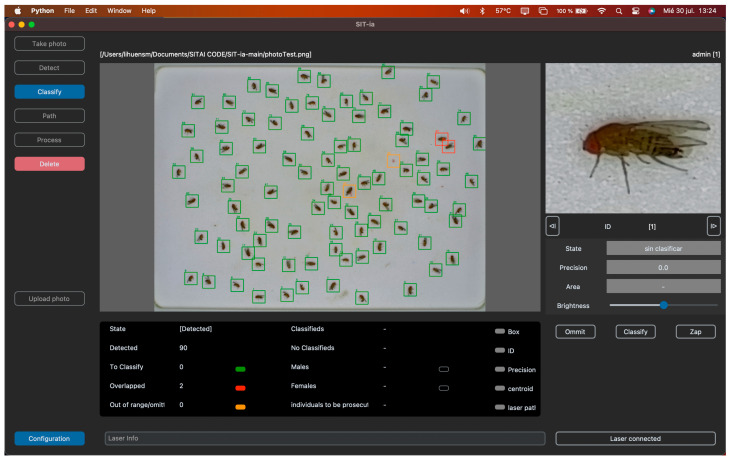
Screen-shot of common processes after detection in SIT-ia: insects ready for classification are marked in green, while overlapping or out-of-bounds individuals were marked in red or orange, respectively. Users can manually label undetected insects or delete unnecessary marks.

The core software component is a Python application allowing operators to manage the entire process, from image capture to laser treatment. This graphical user interface (GUI) was designed for intuitive and efficient interactions. The application has password-protected access. Once authenticated, users are directed to the main screen, where they can begin operating the system. The left panel guides users through each stage of the insect classification process, from image capture to laser elimination of non-useful targeted specimens. In addition to the main screen, a configuration screen allows users to adjust various parameters (i.e., colors). A magnifying tool enables detailed inspection of insects for verification. In normal operation, the process begins with an image capture, reviewed by the user. If the image is suitable, the detection proceeds; otherwise, a new image is captured. During detection, insects ready for classification are marked green, while overlapping or out-of-threshold insects are marked in red or orange, respectively. Users can manually label undetected insects (Figure 1).

In the classification stage, the model labels females in pink and males in blue. Users can review and correct classifications, with corrections stored for future model training. From this point on, user intervention is limited, as the system automatically generates the laser path and eliminates unwanted specimens.

### 3.3. Route Optimization

Three algorithms were evaluated for route optimization, comparing their results to an unoptimized path. The Greedy Algorithm provided a fast nearest-neighbor solution, improving path lengths by 56% compared to unoptimized routes. Local Search further improved results by iteratively refining complete paths, achieving two points greater efficiency than the Greedy approach (58%). The Ant Colony Optimization (ACO) outperformed other methods; it improves by 66% the no-optimization methods through probabilistic path selection guided by “pheromone trails”, dynamically updating optimal routes while avoiding revisits (Table 2).

### 3.4. System Performance

**Figure 2:**
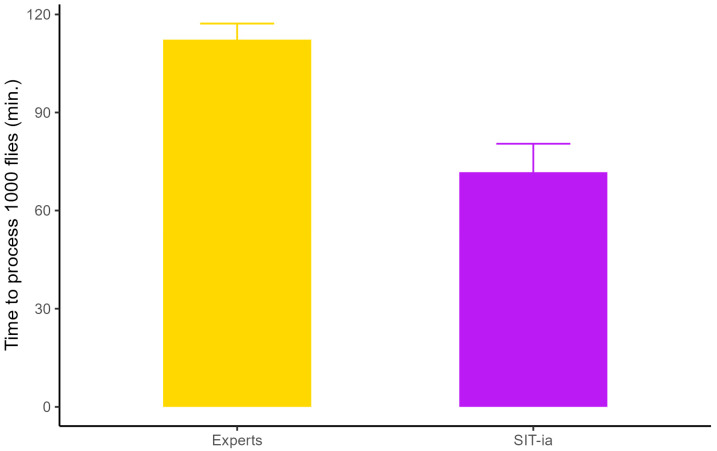
Time expended by experts and the SIT-ia for sex-sorting 1000 flies (including preparation time and sex separation). The orange bar shows the experts’ mean time required to analyze 1000 flies and the purple bar depicts SIT-ia performance. Error bars are the standard error (Wilcoxon test, W = 250, *p*-value = 0.0002).

The sex-sorting performance included the time expended between the preparation of specimens and any corrections that could be made. The strategies of experts (GD, LS, WE, FT, SM) included using light over the plate and using sexual dimorphism (spotted wing in males and ovipositor in females). Only two of the experts resorted to the use of stereomicroscopy. Experts place the anesthetized insects over the plate and at the same time separated flies and sex-sorted them. For the automated system, it is necessary that the flies are separate on the plate to allow individual detection before sex-sorting (Table 3). Another point of comparation of the process is that experts had a negligible error rate (just one error in 7000 individuals) while sorting 1000 flies in batches of 100–300 flies, whereas the automated system has an error rate of <2%. Comparing the time expended by experts and the automated system, we noted that the latter required 71 min (mean= 71.7, SD = 32.5, n = 14) to sort 1000 insects, while experts took 112 min (mean = 112, SD = 22.6, n = 21) (Wilcoxon test, W = 250, *p*-value = 0.0002) (Figure 2).

### 3.5. Explainability

**Figure 3:**
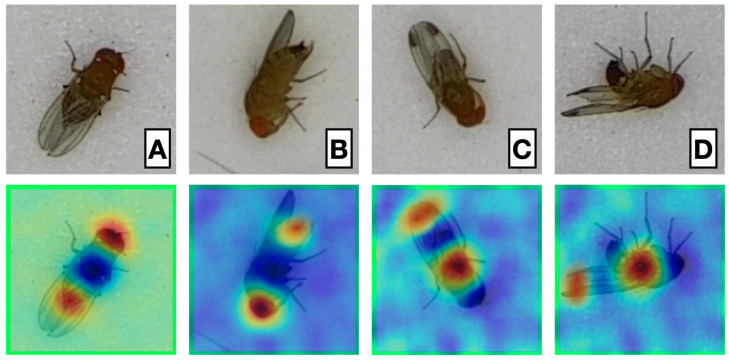
Heat maps generated using the RISE technique (Randomized Input Sampling for Explanation of Black-box Models). Panels (**A**–**D**) display distinct *D. suzukii* specimens, illustrating variations in the model’s attention patterns across different examples. Panels (**A**,**B**) are females, while (**C**,**D**) are males.

The RISE results showed that the model focused on specific features, such as wing spots in males and the serrated ovipositor in females. Unexpectedly, the model also emphasized the abdomen in males (specimens C and D in Figure 3). For specimen D, this could be due to a pronounced curvature characteristic of males when viewed laterally. However, the abdomen of specimen C (also male) was similarly highlighted, suggesting the model identified a distinguishing feature in this body region. Conversely, the model attributed importance to the head in females (specimens A and B), an additional feature used for classification.

## 4. Discussion

The automated system we termed SIT-ia shows the capacity to integrate artificial intelligence and computer vision into Sterile Insect Technique programs. Our results show that automated sex-sorting can achieve high accuracy levels while significantly reducing processing time compared to manual methods. In addition to achieving results 40% faster, performance could be further optimized, as it is currently constrained only by the anesthesia plate size or laser dimensions. While the system remains a prototype, these findings highlight its value as a complementary tool to strengthen SIT implementation. This advancement addresses a critical bottleneck—efficient sex separation—enabling male-only releases to maximize efficacy while minimizing crop damage from residual females [34].

Automated systems for insect sex separation are not new, and it is important to situate our work in this broader context. The International Atomic Energy Agency (IAEA) pioneered efforts in automation for tsetse mass-rearing as early as 2003 [35]. More recently, Zacarés et al. [36] explored the use of computer vision and biometric analysis of pupal size dimorphism to improve sex-sorting in *Aedes* mosquitoes, while Argilés-Herrero et al. [37] developed a Near-InfraRed Pupae Sex Sorter (NIRPSS) capable of reliably distinguishing male and female *Glossina* spp. pupae. Gong et al. [38] further demonstrated the scalability of automated pupal sex-sorting in *Aedes* mosquitoes, providing a robotics-based approach to the mass production of sterile males. In addition, González-Pérez et al. [39] showed that optical sensors coupled with machine learning can accurately classify mosquitoes by genus and sex based on flight characteristics, highlighting the growing role of AI in entomological automation. Complementary to these efforts, Patt et al. [40] described an optical and laser-based system able to identify and selectively eliminate flying insect vectors. Compared to these approaches, SIT-ia offers a distinct advantage: the ability to sort new species without the need for genetic modification, reliance on size pupae dimorphism, or specialized acoustic/optical signatures. Instead, SIT-ia leverages flexible image-based algorithms that can be retrained for a range of pest insects. This adaptability is relevant for pests such as *D. suzukii*, for which sexing strains have yet to be fully developed.

In some cases, SIT programs rely on genetic sexing strains (GSSs) carrying mutations (i.e., temperature-sensitive lethal) or the need for manual or mechanical sorting (i.e., using dimorphism size) [4]. New studies such as that of Liu et al. [12] provide an initial basis for the further optimization of Sexing Element Produced by Alternative RNA-splicing of A Transgenic Observable Reporter (SEPARATOR) for fluorescence-based sex-sorting technology in *D. suzukii*. GSS are not yet available for many other species, and manual sorting is too labor-intensive and may lead to fatigue errors. SIT-ia could help in this issue by automating detection and classification using a custom CNN6 model, which outperformed more complex architectures (e.g., ResNet, VGG) in accuracy and computational efficiency. Although the ACO provided the best solution in terms of path precision, it required 11 times more processing time than the Local Search algorithm. While ACO improved path performance by eight percentual points, its longer computation time made it less practical for time-critical applications. The system’s explainability analysis (via RISE) revealed that the model leverages known morphological features (e.g., male wing spots, female ovipositors) while also identifying secondary traits (e.g., abdominal curvature), suggesting that AI can uncover subtle characteristics that are not apparent to human observers. Although manual sex-sorting for *D. suzukii* has a negligible error rate, our automated system achieved a female contamination rate below 2%, with the added advantage that users can correct the sex of insects before laser annihilation. This performance is comparable with current sexing approaches in other species. Bellini [41] used pupal size-based separation in *Aedes* during a pilot trial and obtained ~1–2% residual females. Automated mosquito sex-sorters also show nontrivial female contamination [42]; separation efficiency in *Anopheles* mosquitoes is limited due to weak dimorphism [43]. Even in other dipterans, such as the American serpentine leafminer (*Liriomyza trifolii*), mechanical sexing only excluded about 76% of females [44]. Although male-only releases are strongly preferable, they are not strictly essential for SIT success, as demonstrated in Lepidoptera and screwworm programs where both sexes are released [5]. However, field trials with *C. capitata* in Guatemala showed that male-only releases were more efficient and cost-effective compared to bisexual releases [34]. For species like *D. suzukii*, where sterile females, if released, continue to cause fruit damage, male-only releases remain crucial. By enabling accurate and efficient male sorting, SIT-ia provides a practical advantage precisely where it is most needed. SIT-ia was designed with modularity in mind, allowing for its straightforward integration into current workflows without major infrastructural changes.

Although industrial-scale SIT programs can produce and release hundreds of millions of sterile insects weekly, eradication programs and suppression strategies could be performed at a smaller-scale, where the number of sterile insects needed could be in the order of 10 thousand a week. As highlighted by Homem et al. [6], Hemer et al. [7] in open tunnels, and Rendón et al. [34], SIT does not always need to operate at landscape or national scales to be effective; a suppression below economic thresholds at farm or regional levels are often enough. Also, there is a trend in soft-skin fruit production to produce under some kind of protection [45], which would even help to maintain the population densities of sterilized males in the crop. The distributed bio-factory model being developed by companies such as BigSis and Senecio shows how SIT can be viable with smaller or modular production units. Within this context, SIT-ia could be deployed in parallel stations to achieve the required throughput for local suppression programs.

Finally, our work reflects a broader trend: the increasing application of AI to entomological research and pest management. Most AI applications have so far focused on monitoring insect populations (e.g., detection on sticky traps); automation is beginning to transform other aspects of SIT as well. Future improvements in computational efficiency, hardware robustness (and safety), and multi-species adaptability will be essential for scaling the proposed technology.

## Figures and Tables

**Table 1:** Neural network architectures used to classify sexes of *Drosophila suzukii* adults. The final dataset comprised 3272 flies, equally split between males and females (1636 each). The data were partitioned into training (60%; n = 1964), validation (20%; n = 654), and test (20%; n = 654) sets, ensuring an equal sex ratio.

Neural Network Architectures Model	Depth (Layers)	Key Architectural Features	Precision (Validation)	Precision (Test)
VGG16	16	Simple stack of 13 convolutional + 3 fully connected layers.	98.27%	97.22%
VGG19	19	Similar to VGG16 but with additional convolutional layers in the first three blocks.	97.40%	96.53%
ResNet50	50	Residual learning blocks with skip connections.	97.83%	97.22%
ResNet101	101	Same as ResNet50 but with more residual blocks.	95.67%	97.22%
ResNet152	152	Same as ResNet50 but with more residual blocks.	95.53%	88.89%
MobileNetV2	~53	Inverted residual blocks with linear bottlenecks.	95.96%	91.67%
EfficientNet	From 237 to 813 (B0–B7)	Uses compound scaling to uniformly balance network width, depth, and resolution.	95.38%	95.83%
CNN6 (Custom)	6	Four convolutional (ReLU) + max-pooling layers, followed by two fully connected layers.	96.95%	98.61%

**Table 2:** Optimization method comparison. Route optimization methods used and improvement against no optimization. Values show mean and standard deviation.

Method	Generation Time [Seconds]	Path Length [Pixels]	Improvement
No Optimization	0.002 (±0.0008)	38,113 (±14,289)	–
Greedy Algorithm	0.29 (±0.08)	30,265 (±8554)	56%
Local Search	0.56 (±0.16)	28,423 (±8033)	58%
Ant Colony	6.58 (±1.86)	22,893 (±6470)	66%

**Table 3:** System performance comparison. Performance of workers (experts and SIT-ia) in different phases of the sorting process for one plate of insects. Values show mean and standard deviation.

Worker	Replicates	Flies per Plate	Preparation (Minutes)	Sex-Sorting (Minutes)
Experts	21	224 (±53)	3.8 (±2.0)	21.3 (±6.9)
SIT-ia	14	123 (±43)	5.8 (±3.4)	2.8 (±2.1)

## Data Availability

The raw data supporting the conclusions of this article will be made available by the authors on request.

## Abbreviations

The following abbreviations are used in this manuscript:

SWD	Spotted Wing Drosophila
SIT	Sterile Insect Technique
ACO	Ant Colony Optimization
AI	Artificial Intelligence
CNN	Convolutional Neural Network
CVPR	Computer Vision and Pattern Recognition
GSS	Genetic Sexing Strain
GUI	Graphical User Interface
PMLR	Proceedings of Machine Learning Research
RISE	Randomized Input Sampling for Explanation of Black-box Models
UAV	Unmanned Aerial Vehicle
VGG	Visual Geometry Group (deep CNN architecture)
IAEA	International Atomic Energy Agency
